# Effects of FOXO3 on Markers of Aging in Blood : An Okinawan Longevity Cohort Study

**DOI:** 10.1093/geroni/igab046.2506

**Published:** 2021-12-17

**Authors:** Richard Allsopp, D Craig Willcox, Trevor Torigoe, Mariana Gerschenson, Michi Shimabukuro, Randi Chen, Tim Donlon, Bradley Willcox

**Affiliations:** 1 Kuakini Medical Center, Honolulu, Hawaii, United States; 2 okinawa International university, International university of Okinawa, Okinawa, Japan; 3 JABSOM, Honolulu, Hawaii, United States; 4 Fukushima University, Fukushima Medical University, Okinawa, Japan; 5 Kuakini Medical Center, Kuakini Medical Center/Honolulu, Hawaii, United States

## Abstract

FOXO3 is among only a few genes that demonstrate a consistently reproducible genetic association with human longevity. We previously demonstrated, in a cross-sectional study of Okinawan Japanese, that the principal longevity variant of FOXO3 (rs 2802292 “G allele”) protects against age-associated attrition of telomere length. We now expand upon this initial observation in a more detailed cross-sectional analysis of the effect of FOXO3 on telomerase activity, FOXO3 expression and inflammatory cytokine levels in both men and women. In agreement with our initial study, we found the FOXO3 longevity variant conferred significant protection against telomere shortening to roughly the same degree in elderly (ages 55 and older) men and women. Carriers of the G - allele also had slightly higher levels of blood telomerase activity in young (ages 20 – 54) and elderly participants (P≤0.1). The expression (mRNA) of FOXO3 increased steadily with age in young and old G – allele carriers (borderline P≤0.08), in contrast to a lack of association with age in non-carriers. The FOXO3 G - allele was also observed to significantly impact levels of both interleukin 6 (IL 6) and IL10, but in a sex dependent manner (P<0.05). These sex-specific effects may point to different mechanisms by which FOXO3 exerts its effect on longevity in men and women.

